# Editorial: Recent Advances in Understanding the Basic Mechanisms of Atrial Fibrillation Using Novel Computational Approaches

**DOI:** 10.3389/fphys.2019.01065

**Published:** 2019-08-20

**Authors:** Jichao Zhao, Oleg Aslanidi, Pawel Kuklik, Geoffrey Lee, Gary Tse, Steven Niederer, Edward J. Vigmond

**Affiliations:** ^1^Auckland Bioengineering Institute, University of Auckland, Auckland, New Zealand; ^2^School of Biomedical Engineering and Imaging Sciences, King's College London, London, United Kingdom; ^3^Department of Cardiology, University Heart Center Hamburg, Hamburg, Germany; ^4^Royal Melbourne Hospital, Melbourne, VIC, Australia; ^5^Department of Cardiology, University of Melbourne, Melbourne, VIC, Australia; ^6^Department of Medicine and Therapeutics, Faculty of Medicine, Chinese University of Hong Kong, Hong Kong, China; ^7^Faculty of Medicine, Li Ka Shing Institute of Health Sciences, Chinese University of Hong Kong, Hong Kong, China; ^8^IMB, UMR 5251, University of Bordeaux, Pessac, France; ^9^IHU Liryc, Electrophysiology and Heart Modeling Institute, Fondation Bordeaux University, Pessac, France

**Keywords:** atrial fibrillation, cardiac electrophysiology, computer simulation, computational modeling, arrhythmia mechanisms

## Where We Are at Regarding Atrial Fibrillation

Atrial fibrillation (AF) is the most common sustained heart rhythm disturbance, associated with substantial morbidity and mortality (Andrade et al., 2014). The current prevalence of AF is ~2% of the general population worldwide and is projected to more than double in the following decades, becoming a global epidemic due to the aging population and the increasing incidence of heart failure and other comorbidities such as hypertension and diabetes (Colilla et al., 2013; Krijthe et al., 2013). Current clinical treatment for AF is suboptimal. Ablation treatment for persistent and permanent AF and AF with concurrent cardiac diseases is disappointing with long term success rates being <30% for single ablation procedures (Brooks et al., 2010; Nishida and Nattel, 2014). Furthermore, anti-arrhythmic drugs (AADs) often lose their efficacy and have side effects (Woods and Olgin, 2014). The poor clinical outcomes are primarily due to a lack of basic understanding of the AF mechanism and quantitative tools to optimize treatment strategies in a clinical setting (Haissaguerre et al., 2007; Hansen et al., 2015).

Novel computational approaches and techniques are playing an important role in our understanding and treatment of AF. Multi-scale computer models of the human atria have been used to investigate the important role of fibrosis in AF and consistently demonstrated that AF is perpetuated by the re-entrant circuits persisting in the fibrotic boundary zones (Bayer et al., 2016; Morgan et al., 2016; Vigmond et al., 2016; Zahid et al., 2016; Zhao et al., 2017). Moreover, models have been applied to propose efficient ablation (Bayer et al., 2016) and AAD (Varela et al., 2016) treatments for AF. To improve patients outcomes, novel computational analysis-aided ablation strategies have also been proposed. Narayan et al. have identified stable AF re-entrant drivers in patients using phase singularity analysis and atrial cellular restitution properties and demonstrated that it was possible to reverse AF in 80.3% of patients by directly targeting these regions in their Focal Impulse and Rotor Modulation (FIRM) trial (Narayan et al., 2014). In addition to the FIRM trial study, Haissaguerre et al. studied 103 patients with persistent AF using a non-invasive ECG imaging (ECGI) approach (Haissaguerre et al., 2014) and concluded that AF is sustained by localized spatially stable drivers where targeted ablation led to 85% of patients being freed from AF at 12 months post ablation. These high success rates are yet to be confirmed in a multi-center randomized clinical trial and the recent REAFFIRM clinical trial presented during a late-breaking session at Heart Rhythm 2019, however, failed to provide evidence of the superiority of the FIRM approach over pulmonary vein isolation. Meanwhile, machine learning is proving to be a promising tool for helping us to understand AF. For example, deep convolutional neural networks have been used to classify AF from single-lead ECGs (Hannun et al., 2019) and to reconstruct 3D left atrial chambers from gadolinium-enhanced MRIs (Xiong et al., 2019) with superior performance.

The aim of this Research Topic was to collect a series of reviews and original research articles presenting recent advances toward a better understanding and treatment of AF through the development or use of: (1) structure-detailed computer modeling; (2) biophysics-based atrial cellular modeling; (3) signal processing and clinical mapping; and (4) meta-analysis and clinical studies. A total of 27 accepted articles were published under this Research Topic. Here in this editorial, we will summarize the new knowledge and approaches generated, and discuss how these can contribute to an improved understanding of AF mechanisms and clinical treatment, as well as how they may shape future research directions.

## Critical Insights Learned From Structure-Detailed Computer Modeling

Improvements in clinical imaging and mapping allow detailed characterization of atrial anatomy, structure and electrophysiology. Computer models of atrial electrical activation provide a powerful computational framework for understanding the structure-function relationship that underlies atrial re-entrant arrhythmias. Atrial structure, including wall thickness, fibrosis, and myofiber orientation, have been suggested to dictate the locations of AF re-entrant drivers in explanted human heart studies (Bishop et al., 2015; Zhao et al., 2015, 2017). Of all atrial structures, fibrosis, the hallmark of structural remodeling, has been investigated extensively in this Research Topic. Clayton studied the effect of the spatial scale (size) of simulated fibrosis on electrical propagations by smoothly varying the diffusion coefficient in 2D atrial tissue models. His study concludes that the spatial scale of fibrosis has important effects on both dispersion of recovery and vulnerability to re-entry. The Aslanidi group evaluated the effects of both atrial wall thickness and fibrosis on AF re-entrant drivers using two sets of computer models, a simple model of an atrial tissue slab with a step change in wall thickness and a synthetic fibrosis patch, and a set of 3D patient-specific computer models based on MRI (Roy et al.). In the slab model, they observed that an AF re-entrant driver drifted toward and along the regions with changes/gradients in wall thickness. Furthermore, they discovered that additional patchy fibrosis would pull the AF re-entrant driver toward it, and that the locations of AF re-entrant drivers were determined by both fibrosis and wall thickness gradients. On the other hand, results from the patient-specific computer models suggested that the interaction between wall thickness and fibrosis plays a very important role in the right atrium due to extensive trabecular structure, whilst fibrosis performs a more decisive role in the left atrium due to a comparably smaller trabecular structure and more extensive fibrotic remodeling (Roy et al.). In another study conducted by Stephenson et al. using micro-CT imaging and anatomically accurate computer modeling, morphological substrates for atrial arrhythmogenesis were discovered in archived human hearts with atrioventricular septal defect. To directly link computer modeling to clinical treatment, Boyle et al. have carried out a multi-modal assessment of the arrhythmogenic propensity of the fibrotic substrate in patients with persistent AF by comparing locations of AF driver regions found in patient-specific computer simulations to those detected by the clinical FIRM approach. They discovered that computer modeling successfully detected most AF driver regions that were identified and ablated using the FIRM approach.

The interaction and impact of atrial structural and electrical remodeling on electrical propagation were also investigated in this Research Topic. The Vigmond group have studied the effects of fibrosis and wavelength on the locations of AF re-entrant drivers using bi-layer atrial models (Saha et al.). They observed that AF re-entrant drivers became more unstable with decreasing wavelength and that driver locations were largely influenced by the degree and distribution of fibrosis as well as the choice of implementation approach. Zhao et al. modeled the loss of lateral connections in atrial myocytes due to fibrotic remodeling and investigated the relative contributions of the sodium and L-type calcium currents to transverse propagation using a simple computer model of two parallel atrial myocyte strands. They discovered that although transverse propagation depends on both sodium and calcium currents, their relative contribution and sensitivity to channel blockage depends on the distribution of transverse connections. Fibrosis is important but structural remodeling involves many factors. Recent experiments suggest that adipocytes lead to a 69–87% increase in action potential duration in neighboring cells as well as an increase in resting membrane potential by 2.5 to 5.5 mV (De Coster et al.). The Panfilov group investigated the electrical interaction of fat and normal myocytes using multi-scale computer models and concluded that adipose remodeling may induce spiral wave dynamics to a complex arrhythmia (De Coster et al.).

Besides, Bueno-Orovio and Ugarte et al. developed a novel approach to model cardiac structural heterogeneity by using a fractional diffusion for the description of cardiac conduction. Their studies remind us that the current cardiac modeling approach itself is not perfect and needs improvement. Dillon-Murphy et al. presented a novel patient-specific modeling workflow for characterizing the thermal-fluid dynamics in the human atria. This is a potentially useful tool for evaluating ablation treatment and minimizing stroke risks.

## Biophysics-Based Atrial Cellular Modeling

The vast majority of patients with AF are treated pharmacologically. However, AADs are often ineffective in ~40% of AF patients (Wyse et al., 2002). Cardiac cellular models were widely used to improve our understanding of electrical remodeling and to facilitate AAD design and development. In this Research Topic, Sutanto et al. presented a novel integrative approach by combining an experimental animal study, confocal imaging and computer modeling to study the effects of the subcellular distribution of ryanodine receptors (RyR2) and L-type Ca^2+^ channels on Ca^2+^ transient properties and spontaneous Ca^2+^ release events (SCaEs) in atrial cardiomyocytes. They discovered that SCaEs preferentially arise from regions of high local RyR2 expression and the propagation of Ca^2+^ waves is modulated by the distance between RyR2 bands. On the other hand, incorporation of axial tubules in various amounts and locations reduce Ca^2+^-transient time to peak; and selective hyperphosphorylation of RyR2 around axial tubules increases the number of spontaneous waves (Sutanto et al.). These novel findings significantly enhance our understanding of the atrial structure-function relationship at the subcellular level. In another modeling study, Colman et al. developed a human atrial cell model derived from a single congruent data source which offers a unique approach for directly relating the model to the experiment.

There are also two important review articles devoted to the modeling of atrial cellular electrophysiology and pharmacotherapy. The Grandi group review recent advances in statistical and computational techniques, i.e., population-based and sample-specific modeling, simulating physiological variability when building cellular computer models of cardiac electrophysiology in both physiological and diseased conditions (Ni et al.); The Koivumäki group detail the unique aspects of AF pathophysiology, modeling approaches for drug testing and how heterogeneity and variability can be incorporated into AF-specific models (Vagos et al.).

## Insights on Signal Processing and Clinical Mapping

Ineffective signal processing and atrial mapping approaches impede our understanding of AF mechanisms and the identification of effective targets for treatment. To determine accurate intracardiac maps, the Rappel group investigated AF re-entrant drivers using phase maps from patients with persistent AF in the presence of various signal contamination (Vidmar et al.). They conclude that domains of low fidelity electrograms can be produced at rotational cores which are most sensitive to far-field activation. By contrast, based on atrial electrograms collected using ECGI from patients with persistent AF, Meo et al. have utilized a new approach to measure AF complexity, a non-dipolar component index, and have correlated this with ablation outcomes and AF pathophysiology. Finally, the Zhang group developed a new 2D convolutional neural network for automatic detection of AF using the MIT-BIH ECG database with superior performance (He et al.).

Animal models and computer simulations are often utilized to validate atrial mapping and signal processing. The Schotten group mapped 12 goats with persistent AF for 3–4 weeks using a 249-electrode array and analyzed the AF episodes collected from the left atrial free wall to quantify its degree of spatiotemporal stationarity (van Hunnik et al.). They discovered that AF properties were stationary; however, they argue that this could not be attributed to stable recurrent conduction patterns. Instead, they postulate that the structural properties of the atria may explain the very variable conduction patterns underlying stationary AF properties. A 64-channel basket mapping catheter was used in the FIRM trials and widely used now in clinics for patients with AF; however, it remains uncertain how reliable this clinical mapping tool is. Alessandrini et al. have developed a computer modeling framework to evaluate basket catheter guided AF ablation. They discovered that a stable re-entrant driver needs a high-density mapping catheter (<3 mm) and a low distance to the atrial surface (<10 mm) for accurate mapping. Finally, the Ganesan group review information theory, such as Shannon entropy, and its application to AF mapping, in the hopes of better pinpointing effective targets (Dharmaprani et al.).

## Meta-Analysis and Clinical Studies

In this Research Topic, there are four original meta-analysis articles. Through a pooled analysis of a total of 17 studies including 5,169 participants, Chen et al. found that the adenosine test and elimination of dormant conduction provoked by adenosine may not improve the long-term success rate in AF patients that undergo circumferential pulmonary vein isolation. Their study raises a serious question about the clinical usage of adenosine to unmask dormant conduction of pulmonary veins as potential reconnection sites. The Tse group has systematically compared AF recurrence rates and complication rates between a novel ablation approach (circular irrigated radiofrequency ablation) and conventional ablation techniques based on 161 original publications (Li et al.). They found that the performance between the two is comparable though circular irrigated radiofrequency ablation has a higher mortality. Filos et al. conducted a scoping review by mapping existing literature in the field of atrial models and their associations with AF to synthesize the vast knowledge toward the mechanism between AF-related P-wave morphologies and atrial computer models. The final meta-analysis study was aided by a novel machine learning approach (Xiong et al.). The growth in medical research publications is accelerating across the board; therefore, there is an urgent need to develop an intelligent automated approach, such as machine learning, to facilitate the identification and selection of relevant articles for meta-analysis. The Zhao group developed a novel machine learning approach to assist in the screening of potentially relevant articles for large-scale meta-analyses and systematic review (Xiong et al.). Their approach led to a 87% reduction in the number of publications needed for manual screening. More importantly, their study demonstrates that diabetes mellitus is a strong, independent risk factor for AF, particularly for women.

It is always important to link or interpret computational approaches and their results back to clinical settings. There are three clinical review papers devoted to this area. Stiles et al. reviewed computational approaches for detecting AF substrates, ranging from complex fractionated atrial electrograms (CFAEs), dominant frequency, ECGI, FIRM, and fibrosis-guided ablation to risk factor modification. Clearly, some of these approaches did not work that well, as demonstrated by recent high-profile clinical studies (Verma et al., 2015), due to our lack of understanding of AF mechanisms. Cheniti et al. focus on reviewing the AF mechanisms that are further obscured and complicated by intermingled multilevel atrial remodeling, various concurrent conditions such as genetic factors (PITX2), obesity/metabolic syndrome, and the limitations of each mapping/imaging/ablation methodology. Bohne et al. systematically review the structural, electrical, and autonomic remodeling underlying elevated AF in diabetes mellitus conditions. Further studies are required to investigate the inter-relationship among obesity, diabetes mellitus, and metabolic syndrome, as well as the role of insulin resistance in AF.

## Conclusions and Future Directions

The articles collected under this Research Topic advance our understanding of atrial structural and electrical remodeling, presenting recent progress on the development of computational modeling, signal processing, atrial mapping, and machine learning approaches, as well as how the gap between basic and clinical studies is being bridged. There is a growing body of evidence supporting a more integrative approach by combining new and established computational and experimental/clinical approaches to improve our understanding and treatment of AF. More importantly, computer modeling of AF will need to be truly multiscale, going from subcellular genetic changes to tissue-level fibrosis to organ-scale geometry and electrical connectivity. AF is a complex disease; therefore, future work should extend the current paradigm to investigate upstream mechanisms and therapy, such as the genetic factors (PITX2) and concurrent clinical conditions (metabolic syndrome). Finally, in the world of meta-data and wearable technology, more effective computational approaches, such as machine learning and large physiological and clinical datasets will need to be used to aid traditional approaches toward further advancements in this exciting research area. Together, these methods will no doubt be an important part of global efforts to tackle this most common, yet elusive, cardiac disease.

## Author Contributions

JZ wrote the draft. The remaining authors provided comments and edits. All authors approved the final version of this article.

### Conflict of Interest Statement

The authors declare that the research was conducted in the absence of any commercial or financial relationships that could be construed as a potential conflict of interest.
